# Numerical comparisons of exponential expressions: The saliency of the base component

**DOI:** 10.3758/s13423-024-02571-8

**Published:** 2024-09-04

**Authors:** Ami Feder, Mariya Lozin, Nadav Neumann, Michal Pinhas

**Affiliations:** Department of Psychology, Ariel University, 4070000 Ariel, Israel

**Keywords:** Exponential expressions, Base-power compatibility, Numerical comparisons, Multi-digit numbers

## Abstract

Exponential expressions represent series that grow at a fast pace such as carbon pollution and the spread of disease. Despite their importance, people tend to struggle with these expressions. In two experiments, participants chose the larger of two exponential expressions as quickly and accurately as possible. We manipulated the distance between the base/power components and their compatibility. In base-power compatible pairs, both the base and power of one expression were larger than the other (e.g., 2^3^ vs. 3^4^), while in base-power incompatible pairs, the base of one expression was larger than the base in the other expression but the relation between the power components of the two expressions was reversed (e.g., 3^2^ vs. 2^4^). Moreover, while in the first experiment the larger power always led to the larger result, in the second experiment we introduced base-result congruent pairs as well. Namely, the larger base led to the larger result. Our results showed a base-power compatibility effect, which was also larger for larger power distances (Experiments 1–2). Furthermore, participants processed the base-result congruent pairs faster and more accurately than the power-result congruent pairs (Experiment 2). These findings suggest that while both the base and power components are processed when comparing exponential expressions, the base is more salient. This exemplifies an incorrect processing of the syntax of exponential expressions, where the power typically has a larger mathematical contribution to the result of the expression.

**Supplementary Information:**

The online version contains supplementary material available at 10.3758/s13423-024-02571-8.

## Introduction

Exponential expressions are used in many fields to describe processes that involve rapid growth and provide a framework for understanding phenomena that follow non-linear patterns, such as population growth and the spread of diseases. Despite their importance, many people struggle to fully comprehend them (e.g., Mullet & Cheminat, 1995; Sastre & Mullet, 1998; Weber, 2002). For example, Weber (2002) found that post-secondary students tended to forget the results of exponential expressions they were presented with and were mostly unable to explain why the results were correct. Similarly, Sastre and Mullet (1998) found that only a small number of high-school and college students estimated exponential expressions correctly when tasked to mark their location on a scale.

Exponential expressions can be categorized under the wider scope of multi-digit numbers which include the integration or computation of multiple digits (Nuerk et al., 2011). This allows us to examine the same cognitive effects found for other multi-digit numbers on exponential expressions. Nevertheless, unlike regular multi-digit numbers, exponential expressions have unique syntactic differences which presumably make their processing more challenging (see also Sastre & Mullet, 1998). In multi-digit numbers, the leftmost digit represents the largest mathematical value, making it easy to compare them by focusing on this digit (Dotan, 2023), while in exponential expressions, the rightmost digit (the power) typically has a larger mathematical contribution to the expression’s result (Sastre & Mullet, 1998). Still, focusing solely on the power component is inadequate. In addition, compared to other mathematical expressions, exponential expressions lack an operand. Moreover, the expressions consist of components with varying sizes and heights. Hence, one needs to decipher them by understanding that the power, although physically smaller than the base, has a larger mathematical role. Given that physical size and numerical value are intertwined (e.g., Feder et al., 2024; Henik & Tzelgov, 1982), this creates a disconnect between these two elements in exponential expressions.

One of the most robust effects studied in multi-digit numbers is the unit-decade compatibility effect (Nuerk et al., 2001). In two-digit number comparisons, faster responses are obtained for unit-decade compatible pairs, where comparisons between tens and units lead to the same decision (e.g., for the pair 34_59, both 3 < 5 and 4 < 9) than for unit-decade incompatible pairs, where comparisons for tens and units lead to different decisions (e.g., for the pair 39_56, 3 < 5, but 9 > 6). The unit-decade compatibility effect is defined as the reaction time (RT) difference between the two conditions. This effect indicates that although only processing of the decades is required to respond correctly, the units cannot be ignored. Another effect that influences multi-digit number processing is the distance effect (Moyer & Landauer, 1967), where the farther apart two numbers are, the faster it takes to compare them (e.g., comparing 2 vs. 9 is faster than 7 vs. 9). In multi-digit number comparisons, there are several distances to consider. For instance, two-digit numbers have a decade, unit, and global distance.

Recently, we adapted the unit-decade compatibility effect to exponential expressions and documented a base-power compatibility effect (Feder et al., 2021), producing faster reactions for base-power compatible pairs, wherein the base and power of one exponential expression were larger than those of the other (e.g., for the pair 3^2^_2^1^, both 3 > 2 and 2 > 1), compared to base-power incompatible pairs, wherein the base and the power led to different decisions (e.g., for the pair 4^1^_3^2^, 4 > 3 but 1 < 2). In this earlier study, we examined automatic processing (i.e., processing that runs without monitoring; Bargh, 1992) of exponential expressions, which is obtained if it occurs although it was not part of the task requirements (Tzelgov, 1997). We utilized the physical size comparison task (Henik & Tzelgov, 1982), in which participants are presented with digits that vary in numerical and physical size, and select the physically larger digit. Reactions are faster for congruent than incongruent trials, where the physically larger digit is also numerically larger (e.g., 1_9 vs. 1_9, respectively). The RT difference between the conditions reflects the size congruity effect (e.g., Henik & Tzelgov, 1982).

Accordingly, in our previous study (Feder et al., 2021) participants were presented with different-sized frames on both sides of the screen, each containing an exponential expression. They were instructed to choose the larger frame. Half the pairs were base-power compatible (i.e., numerically larger base and power in one exponential expression compared to the other), and half base-power incompatible. We found a base-response congruity effect (i.e., faster reactions when the numerically larger base was in the physically larger frame) for both base-power compatibility conditions. However, when we reversed the physical sizes of the components (i.e., a physically larger power compared to the base), we discovered a base-response congruity effect in base-power compatible trials, but a power-response congruity effect (i.e., faster reactions when the numerically larger power was in the physically larger frame) for base-power incompatible trials. These results demonstrated that participants focused on the physically larger component – base or power – suggesting no automatic processing of the correct syntactic structure of exponential expressions.

### The present study

The present study examined participants’ ability to compare exponential expressions intentionally – as required by the task instructions. By focusing on intentional processing, we intended to stir the focus from the physical characteristics of exponential expressions to the mathematical significance of their components, namely, the larger significance of the power component, though it is physically smaller (Sastre & Mullet, 1998). In two experiments, participants were presented with two exponential expressions and were tasked to choose the numerically larger one. We manipulated base-power compatibility, the congruity between the components and the results of the exponential expressions, and the distance between the base and power components. Importantly, while in both experiments half the pairs were base-power compatible and half incompatible, in Experiment 1, the larger power always led to the larger result (i.e., power-result congruent). Thus, it was sufficient for participants to choose the larger power to reach 100% accuracy. In contrast, in the base-power incompatible pairs of Experiment 2, only half of the pairs were power-result congruent (e.g., 2^4^ vs. 3^2^), and in the remaining half, the larger base led to the larger result (i.e., base-result congruent; e.g., 3^4^ vs. 2^5^). Therefore, participants who focused on one component in Experiment 2 (i.e., base or power) would not achieve full accuracy. Importantly, in base-power compatible pairs, whether the participants processed the exponential expressions holistically (i.e., as one unit) or only the base or the power components, they would always compare the exponential expressions accurately.

Our study differed from previous research in several ways. First, we focused on simple exponential expressions, unlike past studies that included negative integers and fractions (e.g., Ebersbach & Wilkening, 2007; Pitta-Pantazi et al., 2007; Weber, 2002). Second, participants were instructed to respond as quickly and accurately as possible, while past studies did not have a direction pertaining to speed. This in turn can drive participants to estimate rather than compute the expressions’ results (e.g., Myachykov et al., 2009), possibly by focusing on certain components of the expressions, automatically or intentionally. Third, our paradigm allowed us to analyze RTs besides accuracy, through which we examined the effects of base-power compatibility, base- and power-result congruity, and distance.

## Experiment 1

### Method

#### Participants

To determine the required sample size, we utilized an analysis of statistical power constructed on a preliminary trial with 15 participants. The power analysis assumed a generalized linear mixed model (GLMM) for RT, treating the participants as a random variable and base-power compatibility, power distance, and base distance as fixed within-participants variables. 200 simulations were executed in R (R Core Team, 2022) via the powerSim function of the *simr* package (Green & MacLeod, 2016). Based on the simulated results, N = 23 participants fulfilled prerequisites of α = .01 and 95% power for the two-way interaction between base-power compatibility and base distance.

Twenty-three undergraduate psychology students (mean age = 23.70 years, SD = 2.46; 18 females, five males; two left-handed; native language: 21 Hebrew, one French, one Spanish) from Ariel University participated in the experiment for course credit. Participants were not diagnosed with dyscalculia or attention-deficit hyperactivity disorder and provided written informed consent before the experiment began. The University’s Institutional Review Board approved the study’s protocol.

#### Apparatus and stimuli

Stimuli were presented using E-Prime 3.0 software (Psychological Software Tools Inc., Pittsburgh, PA, USA) on a 22-in. monitor with a 1,920 × 1,080-pixel resolution. Participants responded by pressing the “A” or “L” keys of a standard QWERTY keyboard with their left and right index fingers, respectively.

The stimuli set included ten pairs of exponential expressions (see Table 1). To keep the expressions relatively simple, the integers of the base and the power components were 2, 3, 4, or 5, and there were no tie expressions (e.g., 2^2^). Moreover, we only included power-result congruent pairs (i.e., the larger power led to the larger result), where half of these pairs were base-power compatible (i.e., the base and the power components in one pair were larger compared to the other), and the other half base-power incompatible (i.e., the base was larger in one pair, and the power was larger in the other). Furthermore, the distance between the base and the power components was 1 or 2. The main motivation behind choosing the specific pairs was to focus on base-power compatibility, creating an equal number of pairs per base-power compatible and incompatible conditions. Unfortunately, because of this, and wanting to keep the pairs rather simple, we could not create a factorial design for the base and power distances as well. For example, in the base-power compatible pairs, we did not have pairs with a distance base of 1 and a power distance of 2, and we only had one pair with a base distance of 2 and a power distance of 1 (Table 1). Each of the ten pairs appeared on both the right and the left sides of the screen and was repeated ten times, giving a total of 200 trials for the experiment.

**Table 1:** Stimuli in Experiment 1 and Experiment 2

Experiment	Base-power compatibility	Base/power-result congruity	Stimuli		Base distance	Power distance
Experiment 1	Base-power Compatible	Base- and power-result congruent	2^3^	3^4^	1	1
			3^2^	4^3^	1	1
			3^2^	5^3^	2	1
			2^3^	4^5^	2	2
			3^2^	5^4^	2	2
			2^3^	3^4^	1	1
	Base-power Incompatible	Power-result congruent	3^2^	2^4^	1	2
			4^3^	3^5^	1	2
			5^2^	4^3^	1	1
			5^2^	3^4^	2	2
Experiment 2	Base-power Compatible	Base- and power-result congruent	2^3^	3^4^	1	1
			3^2^	4^3^	1	1
			2^3^	3^5^	1	2
			2^3^	4^5^	2	2
			3^2^	5^3^	2	1
			3^2^	5^4^	2	2
	Base-power Incompatible	Base-result congruent	3^4^	2^5^	1	1
			4^2^	2^3^	2	1
			5^3^	3^4^	2	1
		Power-Result Congruent	3^2^	2^4^	1	2
			4^3^	3^5^	1	2
			5^2^	3^4^	2	2

Numbers were presented in black Calibri font on a grey background. The base component was presented in 138-pt font size, and the power component in 100-pt font size.

#### Procedure

Each participant was tested individually in a quiet laboratory room. The participants sat in front of a computer screen, with their index fingers placed on the keyboard’s response keys. Each trial began with a centered fixation cross for 500 ms, followed by a pair of exponential expressions, which remained visible until response. After a response was made, a 500-ms interval of a blank screen was presented before the next trial. Trials were randomly ordered. Participants were asked to choose as quickly and as accurately as possible the numerically larger exponential expression. If the larger expression was on the left/right side of the screen, they were asked to press the “A”/“L” key, respectively. A short practice of ten trials was presented before the task began to make sure participants understood the task. After every 50 trials, a short self-paced break was presented, also providing feedback on mean accuracy and RT. The experiment lasted ~15 min.

#### Analysis

All data were analyzed using IBM SPSS Statistics (Version 26). In all experiments, we excluded RTs that were ± 2.5 SD of each participant’s individual mean (< 1% of the data per experiment). The RT analysis was conducted on correct responses only.

Several considerations guided our statistical analyses. First, the design was not factorial, which omitted the option of a full factorial model. Second, our main interest was to examine the effects of base-power compatibility for all possible combinations of base and power distances. However, because base and power distances were confounded in our design, we did not examine the three-way interaction between base-power compatibility, base distance, and power distance. Third, since both base and power distance covariates comprised two levels, the probability for multicollinearity in GLMMs between effects that include the same covariate (e.g., a main effect of base distance and an interaction between base distance and power distance) is rather high. Therefore, base/power distance covariates were not included as multiple effects in the same model.

Accordingly, in both experiments we conducted three separate GLMMs using random intercepts for both mean RTs of correct responses (~92% of the data) and mean error rates (ER; ~99% of the data). All GLMMs included base-power compatibility (compatible, incompatible), base distance (1, 2), and power distance (1, 2) as fixed (within-participants) factors, and participants as the random factor. In the first analysis, we examined the main effect of base-power compatibility while both base and power distances were entered as covariates. In the second analysis, we examined the interaction between base-power compatibility and base distance, while power distance was entered as a covariate. In the third analysis, we examined the interaction between base-power compatibility and power distance, while base distance was entered as a covariate. Lastly, for both experiments we conducted an additional GLMM analysis which included only pairs with fixed base and power distances (i.e., base distance and power distance were identical). This analysis included base-power compatibility (compatible, incompatible), and a new variable of fixed distance (1, 2) as factors (see Online Supplementary Material (OSM), Figs. S1 and S2).

In all the analyses, we used a log-likelihood ratio test to compare the GLMM to a reduced GLMM in which the factor/s in question were removed to ascertain whether the effect of a specific factor/s were significant. We provide the degrees of freedom, the p-value, and the test statistic (LL1 – LL0), which follows a χ^2^ distribution (LL0 and LL1 stand for the log-likelihoods of the reduced and full models, respectively).

The experiment and analyses were not preregistered. The data and materials of this study are available via the Open Science Framework at: https://osf.io/q3w95/?c9515addbf7947de95cc600847dab894.

### Results and discussion

**Fig. 1:**
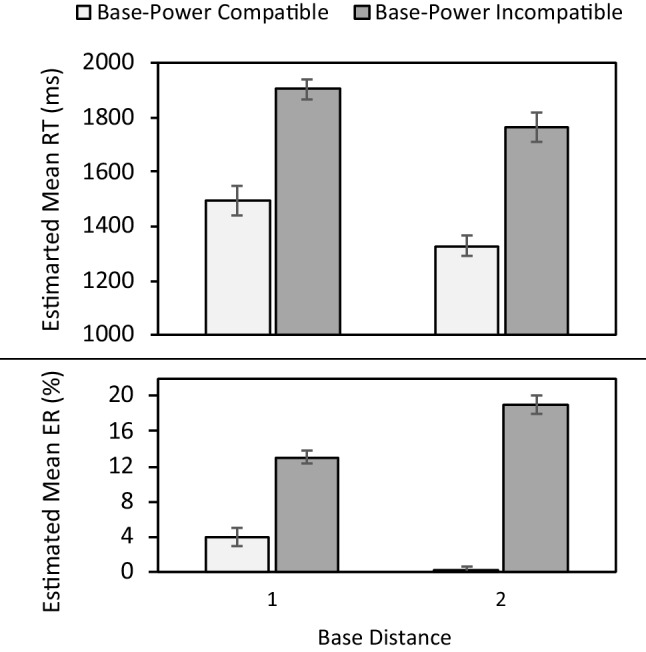
Estimated mean reaction time (RT) (upper panel) and error rate (ER) (lower panel) in Experiment 1 as a function of base-power compatibility and base distance. Error bars denote -/+ 1 SE

**Fig. 2:**
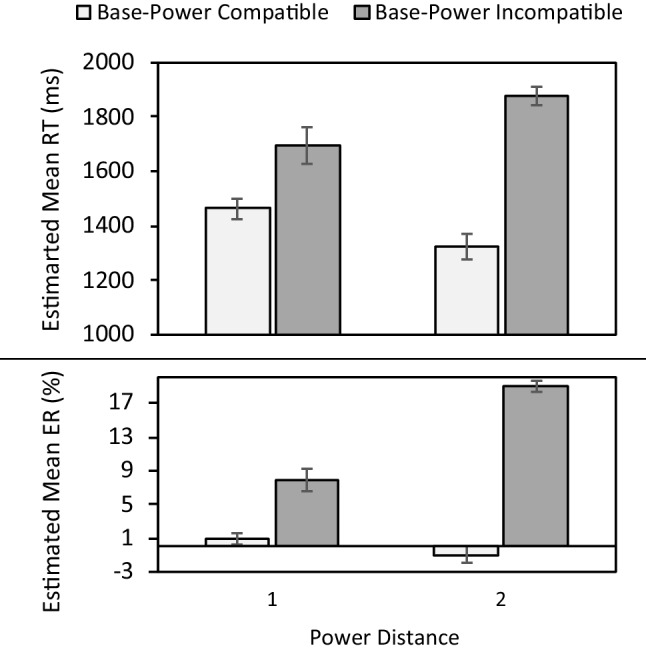
Estimated mean reaction time (RT) (upper panel) and error rate (ER) (lower panel) in Experiment 1 as a function of base-power compatibility and power distance. Error bars denote -/+ 1 SE

Both RT and ER analyses revealed a significant main effect for base-power compatibility (RT: χ^2^(1) = 75.89, *p* < .001; ER: χ^2^(1) = 240.84, *p* < .001), demonstrating slower RTs (1,834 vs. 1,408 ms) and higher ERs (16% vs. 1%) for base-power incompatible compared to compatible trials. Moreover, significant Base-Power Compatibility × Base Distance interactions (RT: χ^2^(2) = 138.27, *p* < .001; ER: χ^2^(2) = 309.86, *p* < .001; Fig. 1) revealed a larger base-power compatibility effect for base distance 2 (RT: *M =* 438 ms, *p* < .001; ER: *M =* 19%,* p* < .001) compared to 1 (RT: *M =* 410 ms, *p* < .001; ER: *M =* 9%,* p* < .001). Similarly, significant Base-Power Compatibility × Power Distance interactions (RT: χ^2^(2) = 116.03, *p* < .001; ER: χ^2^(2) = 483.85, *p* < .001; Fig. 2) revealed a larger base-power compatibility effect for power distance 2 (RT: *M =* 550 ms, *p* < .001; ER: *M =* 20%,* p* < .001) compared to 1 (RT: *M =* 230 ms, *p* = .002; ER: *M =* 7%,* p* < .001).

The base-power compatibility effect showed faster processing when both base and power components were larger in one expression than the other compared to when only one component was larger. This is consistent with the unit-decade compatibility effect (Nuerk et al., 2001), where both components are processed. Crucially, while the unit digits can be ignored to reach a sufficient decision in between-decade two-digit number comparisons (Ganor-Stern et al., 2007), ignoring the base component completely in exponential expressions would not be a wise strategy altogether. The base plays a role in the result of the expression, particularly in our stimuli set, in which half of the pairs were base-power compatible (e.g., for the pair 4^5^_3^2^, both 4 > 3 and 5 > 2), which means they were base- and power-result congruent. Therefore, we cannot surmise whether participants processed the exponential syntax correctly, or whether they focused more on a specific component. Alternatively, the finding of a base-power compatibility effect may stem from the distance between the results of the compared exponential expressions. The average distance between the expressions’ results was smaller for base-power incompatible (*M* = 79.8) compared to compatible (*M* = 375.2) trials. According to the distance effect, this should result in faster and more accurate reactions for compatible trials, which was indeed the case.

Moreover, the interactions between base-power compatibility and components’ distances indicate that larger distances between the base/power made it easier to process inconsistencies between the components, leading to a stronger base-power compatibility effect. Importantly, we kept the componential distance which was not included in the effect as a covariate in our model to ensure that each component's processing was independent of the other.

Together, the findings of Experiment 1 indicate the processing of both base and power components. However, because all pairs were power-result congruent, we could not determine which component was more salient during exponential expressions’ processing. Experiment 2 attempted to tackle this question by incorporating base-power incompatible pairs that were base-result congruent.

## Experiment 2

### Method

#### Participants

Twenty-four undergraduate psychology students (mean age = 22.38 years, SD = 2.60; 22 females, two males; two left-handed; native language: 21 Hebrew, two English) from Ariel University participated in the experiment in exchange for course credit.

#### Apparatus and stimuli

The stimuli were the same as in Experiment 1, except for the fact that in this experiment, we included power-result *incongruent* pairs as well (i.e., base-result congruent pairs) (Table 1). The design included 12 pairs of exponential expressions; six were base-power compatible, where five of them were identical to the pairs in Experiment 1 (we added another pair so we could have an even number of stimuli for each base/power-result condition); six were base-power incompatible: three were power-result congruent, and three base-result congruent, therefore, we referred to them as such. Due to the limits of the design (for a detailed discussion of this point see *Apparatus and stimuli* section of Experiment 1), for base-power incompatible pairs, we were unable to include a power distance of 2 for base-result congruent pairs and a power distance of 1 for power-result congruent pairs. Moreover, in base-power compatible pairs, we had only one pair with a base distance of 2 and a power distance of 1, and one pair with a base distance of 1 and a power distance of 2. In base-power incompatible pairs, there was only one base-result congruent pair with base and power distances of 1, and only one power-result congruent pair with base and power distances of 2. In total, there were 240 trials for the experiment (i.e., 12 pairs × 2 left/right side × 10 repetitions = 240).

#### Procedure

The procedure was identical to Experiment 1, besides presenting the self-paced breaks after every 60 trials.

#### Analysis

We conducted the same three GLMM analyses for the mean RTs of correct responses (~85% of the data) and mean error rates (> 99% of the data) as in Experiment 1. These analyses included base-power compatibility (compatible, incompatible), base distance (1, 2), and power distance (1, 2) as (fixed) factors. Hence, in these analyses, we did not separate between the two types of base-power incompatible pairs (i.e., base-result congruent and power-result congruent) to ease the comparability of both experiments’ findings. Then, to test for the possible impact of base/power-result congruity, we conducted a further GLMM analysis on the mean RT for correct responses and the mean ER with pair type as a factor. Since we were only interested in examining the difference between the two base-power incompatible pairs, we only include base-result congruent, and power-result congruent pairs as levels of the pair type factor.

All other details of the methodology were identical to Experiment 1, including the GLMM analysis with fixed distances described in the OSM.

#### Results and discussion

**Fig. 3:**
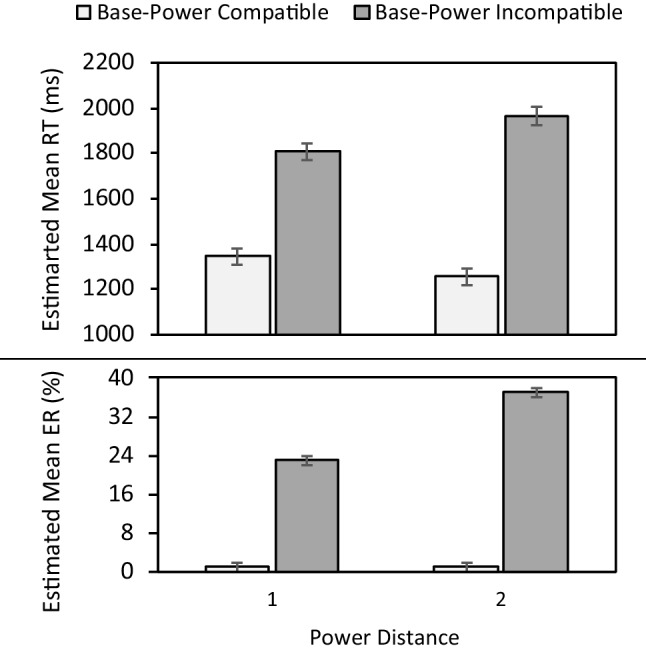
Estimated mean reaction time (RT) (upper panel) and error rate (ER) (lower panel) in Experiment 2 as a function of base-power compatibility and power distance. Error bars denote -/+ 1 SE

Replicating the findings of Experiment 1, significant main effects of base-power compatibility (RTs: χ^2^(1) = 240.37, *p* < .001; ER: χ^2^(1) = 1,115.01, *p* < .001) demonstrated slower RTs (1,878 vs. 1,300 ms) and higher ERs (30% vs. 1%) for base-power incompatible compared to compatible trials. Moreover, significant Base-Power Compatibility × Power Distance interactions (RT: χ^2^(2) = 251.07, *p* < .001; ER: χ^2^(2) = 1,243.79, *p* < .001; Fig. 3), revealed a larger base-power compatibility effect for power distance 2 (RT: *M =* 709 ms, *p* < .001; ER: *M =* 36%,* p* < .001) compared to 1 (RT: *M =* 462 ms, *p* < .001; ER: *M =* 22%,* p* < .001). This again exemplifies that both components were processed, as well as the fact that stronger base-power compatibility was achieved by processing inconsistencies between the components more easily at larger distances between the base and power. However, the Base-Power Compatibility × Base Distance interactions revealed speed-accuracy trade-offs (see OSM, Fig. S3).

Importantly, the analysis contrasting both types of base-power incompatible pairs revealed a significant main effect (RTs: χ^2^(1) = 7.55, *p* = .006; ER: χ^2^(1) = 73.37, *p* < .001), demonstrating faster and more accurate reactions for base-result congruent pairs compared to power-result congruent pairs (RTs: 1,788 vs. 1,983 ms; ER: 23% vs. 38%).

Collectively, these findings indicate that participants focused more on the base component, aligning with Feder et al.'s (2021) data showing the saliency of the physically larger component (base or power) during comparisons. Such findings suggest processing based on physical size rather than exponential syntax. In the current study, participants might have been expected to focus on the power component due to its larger mathematical significance (Sastre & Mullet, 1998). Alternatively, if participants processed both base-power incompatible conditions similarly, it would suggest they focused on the expressions results or on both components equally, which could have been an appropriate strategy.

## General discussion

Given that people generally struggle with understanding exponential expressions (e.g., Mullet & Cheminat, 1995; Sastre & Mullet, 1998; Weber, 2002) and we instructed participants to compare such expressions quickly, we hypothesized they would estimate rather than calculate the expressions. Hence, we expected participants would focus more on one component rather than process the whole expression. The results showed that participants focused on the base – the “less relevant” component – producing a base-power compatibility effect.

In comparisons of two-digit numbers from different decades, though processing the unit digits is not necessary for a decision regarding the larger number, they cannot be ignored, as apparent by the unit-decade compatibility effect (Nuerk et al., 2001). Nevertheless, in such comparisons, participants focus more on the decade digits, which have larger mathematical significance (e.g., Fitousi & Algom, 2020). In contrast, in exponential expressions, participants focus more on the mathematically less significant component – the base. Importantly, though we ensured in our design that focusing on the base component would be less relevant for the task, we found faster and more accurate processing for base-result congruent pairs than for power-result congruent ones.

Because the base-power compatibility effect was larger for greater power (and base in Experiment 1) distances, it indicated that participants also processed the power component. Experiment 2 replicated this effect, adding pairs where the larger base led to the larger result. This inclusion allowed us to determine which component was more salient during processing. We found that participants processed base-result congruent pairs (e.g., for the pair 3^4^_2^5^, base 3 > 2 and the result 81 > 32) more quickly and accurately than power-result congruent pairs (e.g., for the pair 3^2^_2^4^, base 3 > 2 but the result 9 < 16), indicating the base was more salient.

Why did the physically larger element take precedence over syntax? When addressing the stimuli’s physical size is required by the task, as occurred in Feder et al. (2021) where participants chose the physically larger component, this is to be expected. This presumably shifted participants’ attention towards the physically larger elements, making the physically larger digits more salient. Contrarily, here, participants were tasked to focus on the numerical entities. One possible explanation can therefore be the uniqueness of exponential expressions – mathematical entities where digits vary in size. Importantly, numbers are inseparable from their physical elements (e.g., Leibovich et al., 2017), specifically size. As evidenced by the size congruity effect, physical size is strongly associated with numerical size (Henik & Tzelgov, 1982) and place value (e.g., Feder et al., 2024). Hence, it is possible that participants associate the physically larger component (i.e., the base) with a larger result, and therefore, focus more on that component. Alternatively, participants may focus on the larger element regardless of the task because of its physical saliency. Given that participants were meant to execute the task as quickly as possible, they were limited in time to process all elements of the exponential expression. Deriving from findings showing that physical size is processed before nonsymbolic numerical information (e.g., Leibovich et al., 2017), we can conjecture that participants are drawn to the physically larger base first, and only then process that element numerically.

It also may be that physicality is not a factor. It is possible that the base was salient for other reasons. For example, its centrality on the screen compared to the power, or possibly a left-digit bias arising from the place value structure of number syntax, where the leftmost digit is numerically the largest (Dotan & Dehaene, 2020). Uniquely, the syntactical structure of exponential expressions is reversed from multi-digit numbers in that manner. However, when controlling for these elements previously in physical comparisons of exponential expressions (Feder et al., 2021), the component’s physical size remained the dominant factor. Yet, considering there may be other processes involved during intentional processing of exponential expressions, we cannot completely negate these alternatives.

## Limitations and future directions

Despite the novelty and contribution of the current findings, they have limitations. First, to maintain an equal number of base-result congruent and power-result congruent pairs in Experiment 2, we narrowed our selection of power distance levels, limiting our ability to examine specific componential distance effects. Additionally, we only included two distances between bases and powers, creating a potential confound between base and power distances and compatibility. Nevertheless, by entering the distance factors as covariates, we ensured participants’ preference for the base was not due to uneven stimuli representation. Future research can expand the componential distances tested to gain further insight into distance effects on exponential expression processing.

A second limitation is that the power is physically smaller than the base. Our current and previous (Feder et al., 2021) findings indicate that physical size appears more influential in processing exponential expressions than syntax. Future research can experiment with different syntactic forms to “level the physical playing field” for both components of exponential expressions.

Lastly, while we asked participants to compare the expressions quickly and accurately, we did not examine whether their focus on a specific component was intentional or automatic, nor whether these strategies varied between or within participants and trials. Further research can explore these strategies to better understand how exponential expressions are processed.

## Conclusion

In summation, participants processed both components of the exponential expressions. However, when both components are pinned against each other, the base is more salient during processing. This displays an incorrect syntactic understanding of exponential expressions, focusing on the less mathematically impactful component. Amalgamating the findings of this study and Feder et al. (2021), due to the importance of exponential expressions and that they represent an essential mathematical concept, additional research is necessary to devise novel methods and strategies to better handle them. This could potentially simplify the comprehension of exponents and exponential growth, especially given the inherent disconnect between physical size and mathematical significance in exponential expressions.

## Supplementary Information

Below is the link to the electronic supplementary material.

Supplementary file1 (DOCX 233 KB)

## Data Availability

Materials and data are available via the Open Science Framework at https://osf.io/q3w95.
