# Supplementary material for: Numerical comparisons of exponential expressions: The saliency of the base component
Source: Psychon Bull Rev. 2024 Sep 4;32(2):705–13. doi: 10.3758/s13423-024-02571-8 (PMC12000108; doi:10.3758/s13423-024-02571-8)
Supplement: Supplementary file 1 — Supplementary file1 (DOCX 233 KB) [file 13423_2024_2571_MOESM1_ESM.docx]

**Supplementary Material**

*Figure S1*. Estimated mean RT (upper panel) and ER (lower panel) in Experiment 1 as a function of base-power compatibility and fixed distance. Error bars denote -/+ 1 SE.
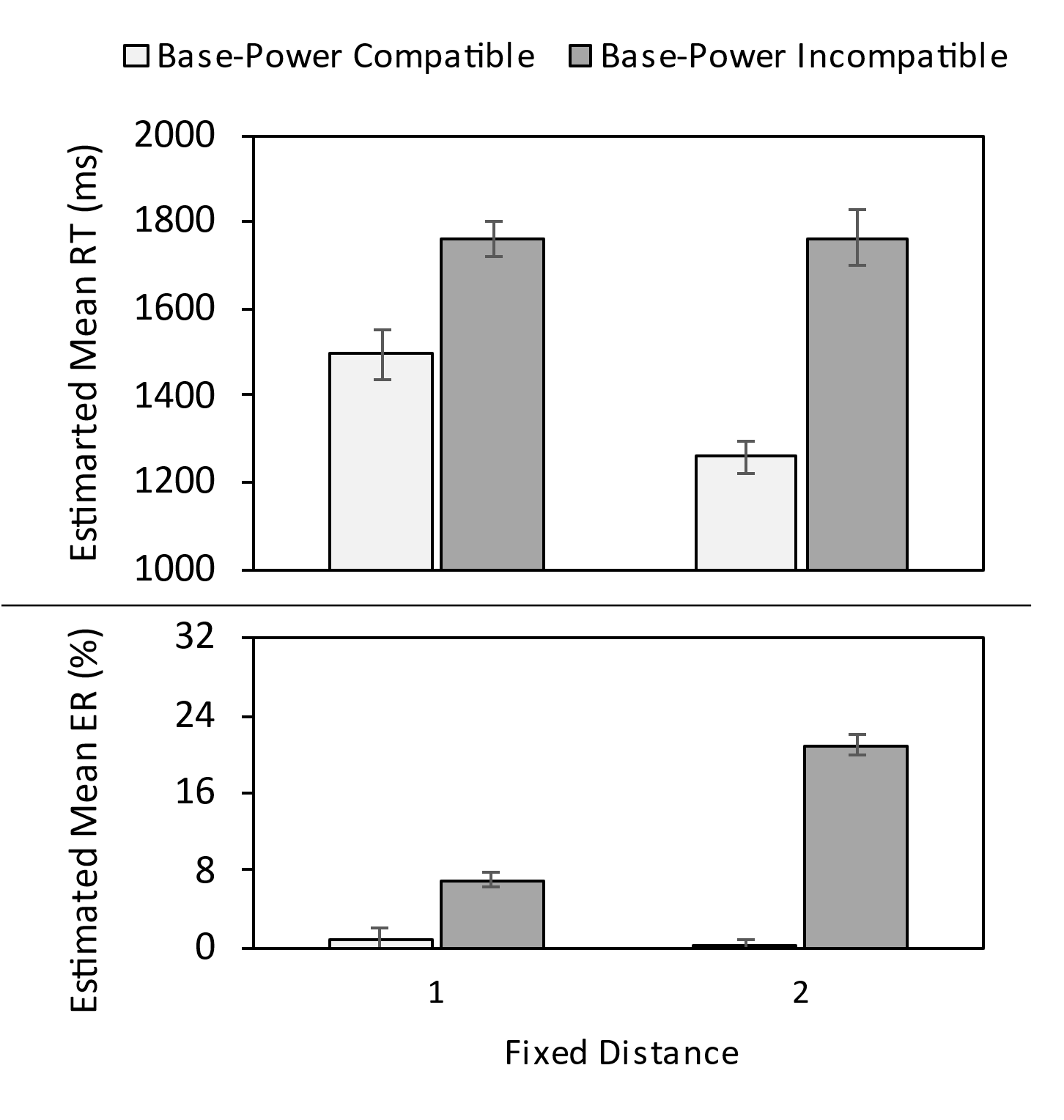


Both RT and ER analyses revealed a significant main effect for base-power compatibility (RT: χ2(1) = 67.09, *p* < .001; ER: χ2(1) = 221.37, *p* < .001), demonstrating slower RTs (1,763 vs. 1,378 ms) and higher ERs (14% vs. 1%) for base-power incompatible compared to compatible trials. Moreover, a significant Base-Power Compatibility × Fixed Distance interaction (RT: χ2(3) = 17.44, *p* < .001; ER: χ2(2) = 99.96, *p* < .001) revealed a larger base-power compatibility effect for a fixed distance of 2 (RT: *M* = 505 ms, *p* < .001; ER: *M* = 21%, *p* < .001) compared to 1 (RT: *M* = 265 ms, *p* < .001; ER: *M* = 6%, *p* < .001).

*Figure S2*. Estimated mean RT (upper panel) and ER (lower panel) in Experiment 2 as a function of base-power compatibility and fixed distance. Error bars denote -/+ 1 SE.
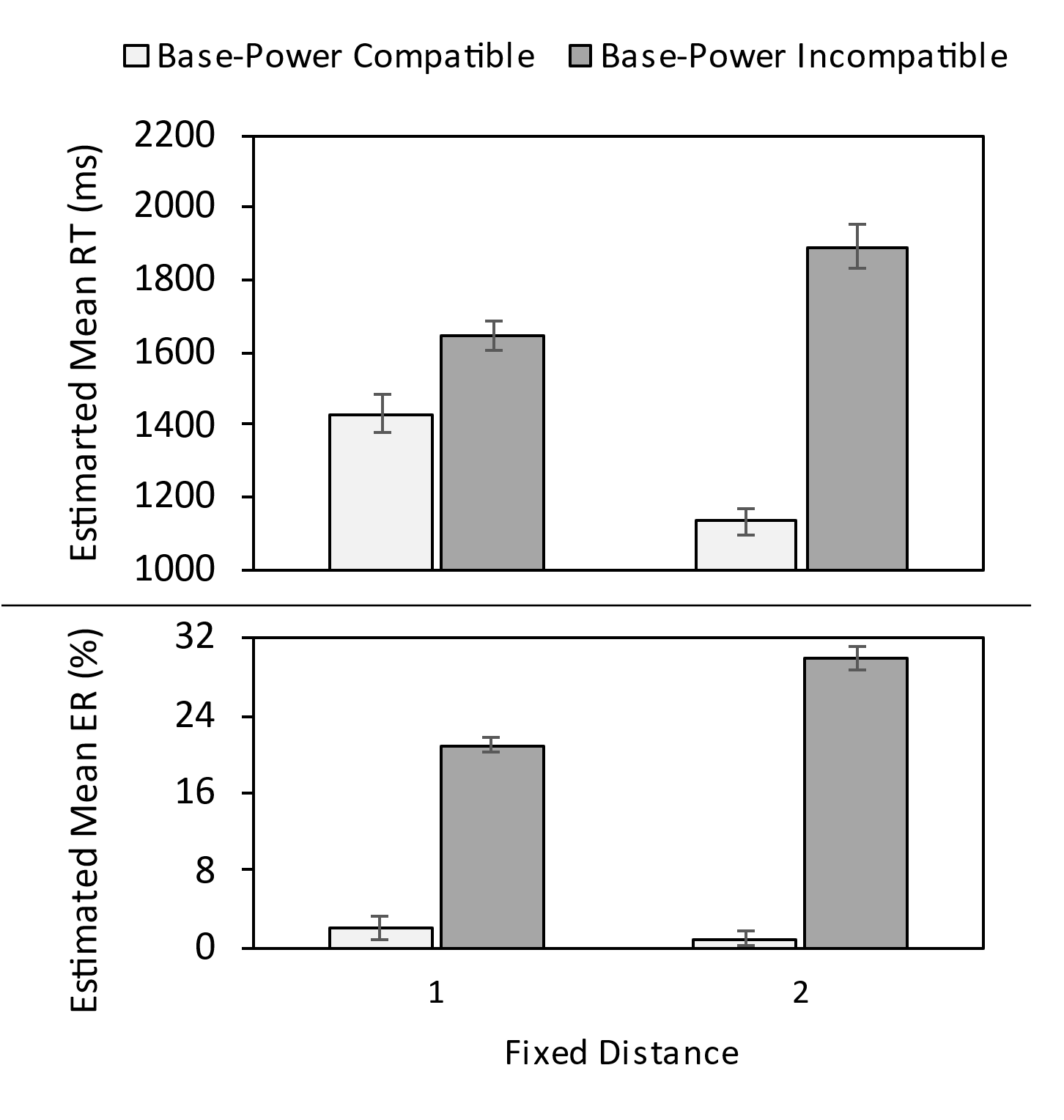


Both RT and ER analyses revealed a significant main effect for base-power compatibility (RT: χ2(1) = 93.83, *p* < .001; ER: χ2(1) = 548.04, *p* < .001), demonstrating slower RTs (1,768 vs. 1,282 ms) and higher ERs (26% vs. 1%) for base-power incompatible compared to compatible trials. Moreover, a significant Base-Power Compatibility × Fixed Distance interaction (RT: χ2(2) = 41.49, *p* < .001; ER:, χ2(2) = 28.90, *p* < .001) revealed a larger base-power compatibility effect for a fixed distance of 2 (RT: *M* = 759 ms, *p* < .001; ER: *M* = 30%, *p* < .001) compared to 1 (RT: *M* = 213 ms, *p* < .001; ER: *M* = 20%, *p* < .001).

*Figure S3*. Estimated mean RT (upper panel) and ER (lower panel) in Experiment 2 as a function of base-power compatibility and base distance. Error bars denote -/+ 1 SE.
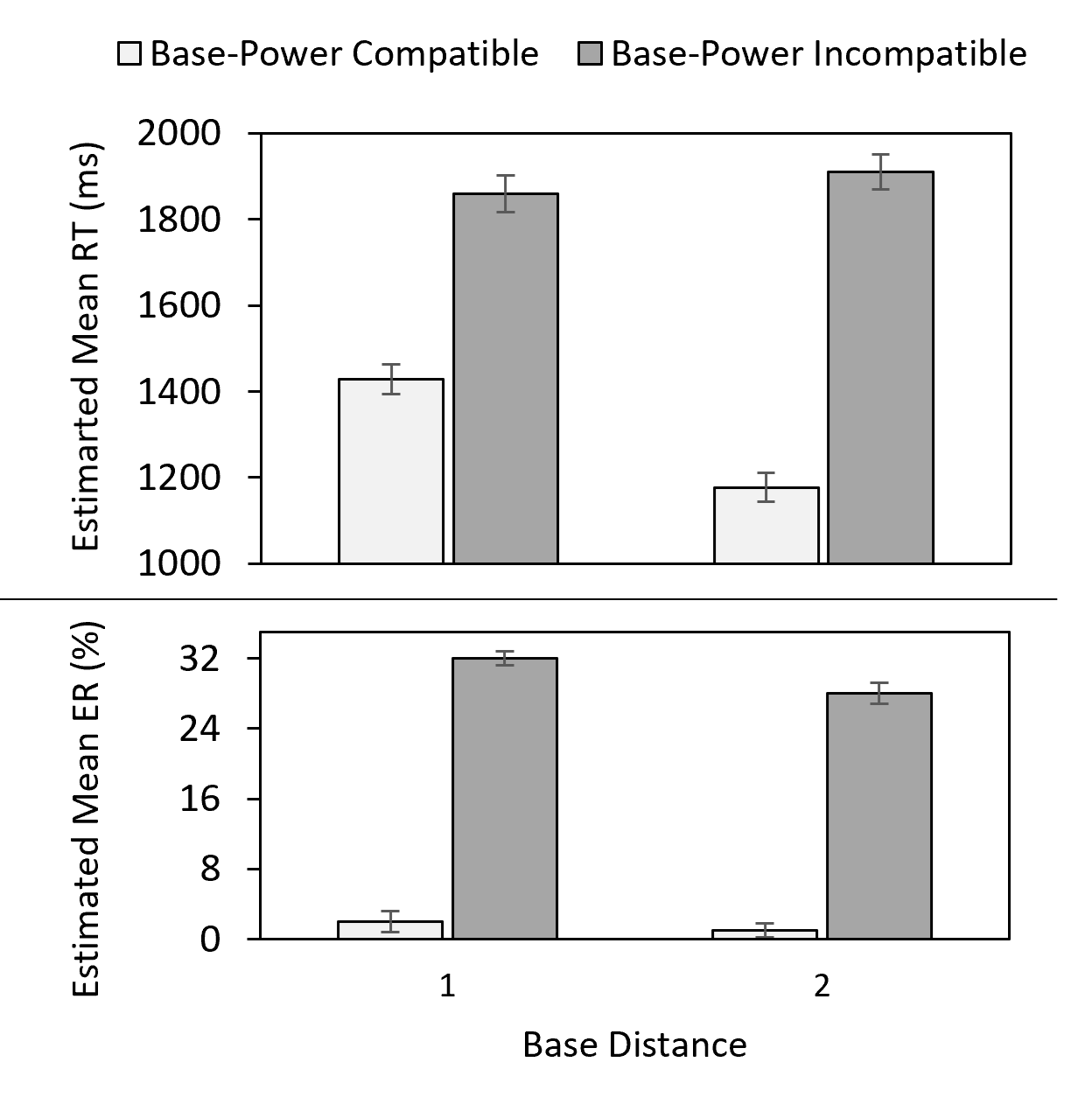


Significant Base-Power Compatibility × Base Distance interactions (RT: χ^2^(3) = 270.49, *p* < .001; ER: χ^2^(3) = 1,148.45, *p* *<* .001) revealed a larger base-power compatibility effect for a base distance of 2 (*M =* 722 ms, *p* < .001) compared to 1 (*M =* 427 ms, *p* < .001) in the RT analysis, but a smaller base-power compatibility effect for a base distance of 2 (*M =* 28%, *p* < .001) compared to 1 (*M =* 31%, *p* < .001) in the ER analysis, presenting a speed-accuracy tradeoff. Therefore, we cannot discuss the findings of this effect.
